# Live cell quantum multiphysiology enabled by a manipulable single nanodiamond

**DOI:** 10.1093/nsr/nwaf130

**Published:** 2025-04-01

**Authors:** Yang Xu, Yibo Yang, Yuang Chen, Wenxin Zhu, Shiyang Lyu, Chen Zhang, Xingxu Huang, Jiandong Feng

**Affiliations:** Laboratory of Experimental Physical Biology, Department of Chemistry, Zhejiang University, Hangzhou 310058, China; Laboratory of Experimental Physical Biology, Department of Chemistry, Zhejiang University, Hangzhou 310058, China; Laboratory of Experimental Physical Biology, Department of Chemistry, Zhejiang University, Hangzhou 310058, China; Laboratory of Experimental Physical Biology, Department of Chemistry, Zhejiang University, Hangzhou 310058, China; Laboratory of Experimental Physical Biology, Department of Chemistry, Zhejiang University, Hangzhou 310058, China; Laboratory of Experimental Physical Biology, Department of Chemistry, Zhejiang University, Hangzhou 310058, China; The First Affiliated Hospital, School of Medicine, Zhejiang University, Hangzhou 310003, China; Laboratory of Experimental Physical Biology, Department of Chemistry, Zhejiang University, Hangzhou 310058, China; The First Affiliated Hospital, School of Medicine, Zhejiang University, Hangzhou 310003, China; Institute of Fundamental and Transdisciplinary Research, Zhejiang University, Hangzhou 310058, China

**Keywords:** NV centers, intracellular measurement, nanodiamond, quantum sensing, multiphysiology

## Abstract

The patch-clamp technique provides direct insight into electrophysiology. However, probing multiphysiology such as local temperature and electromagnetic field dynamics within a single live cell remains a challenge. Here we report live cell quantum multiphysiology using a manipulable-single-nanodiamond-based electron spin sensor. By electrically trapping and integrating a single nanodiamond onto a glass nanopipette, we achieve its 3D manipulation within a single live cell. This enables the first nanoscale controlled live cell quantum sensing of multiphysiology signals under native conditions, providing direct insight into localized intracellular activities. Our observations reveal a spatial heterogeneity in temperature over different sites within a single cell, indicative of local activity-induced heat production. Furthermore, temporally resolved relaxation measurements effectively capture the intracellular free-radical-mediated local electro-magnetic noise dynamics. We find a number of unexpected fluctuation phenomena of electromagnetic noise that may directly represent different types of intracellular free radical generation dynamics. Live cell quantum multiphysiology bridges the gap between high-physical-sensitivity-quantum sensing with high-spatiotemporal-resolution live cell measurements, opening up a new avenue for accessing cellular function with unprecedented capabilities.

## Teaser

As an analogy to ‘Patch clamp’ techniques, the authors developed a ‘quantum patch clamp’ technique based on manipulating a single nanodiamond for performing quantum multiphysiology inside a single live cell.

## INTRODUCTION

Single-cell and single-molecule technologies have revolutionized our understanding of cell functions by providing direct insight into cellular complexity, heterogeneity and dynamics [1,2]. An outstanding goal is to probe, *in situ*, the precise dynamics at exactly the time and place that the physiological reaction occurs [3,4]. Toward this goal, it is essential to directly measure the multiphysical outputs of the local intracellular processes, encompassing spatiotemporally resolved temperature, force, and electromagnetic field within a single live cell, namely cell multiphysiology. The patch-clamp technique [5] has provided a direct and effective methodology for intracellular electrophysiology but multiphysiology remains unfulfilled. This highlights the necessity for developing new techniques capable of measuring multiphysical quantities. The response of conventional functional fluorescence probes [6,7] cannot simultaneously record multiphysical signals and is also limited by sensitivity. On the other hand, quantum sensing can now directly probe multiphysical signals with ultrahigh sensitivity [8–10]. Due to the atomic length scale, the long coherence time at room temperature, and the physical quantity sensing capability, nitrogen-vacancy (NV) center-based quantum sensors [11] have shown proof-of-concept uses in biological systems [12–17]. Despite their atomic feature, practically delivering nanoscale NV quantum sensing into live cells necessitates a spatially controlled and confined probe–target interaction space [9]. Current approaches for bringing the NV sensor to the measurement object are mainly based on interfacial imaging with bulk diamond chip [13] and localized sensing with atomic force microscopy (AFM) [18,19] or nanodiamonds [12,20,21]. However, the live cell environment is inherently complex, delicate and dynamic. The lack of active and precise delivery and manipulation of single quantum objects [22,23] under biological conditions greatly hinders controllable quantum sensing inside a single cell. Spatiotemporally resolved live cell quantum multiphysiology remains elusive.

Here we develop a manipulable-single-nanodiamond (MSN) probe-based ‘quantum patch clamp’ for live cell quantum multiphysiology, an analogy to the patch-clamp technique [5] in single-cell electrophysiology for single-channel current recording. Our key to actively manipulating a quantum probe inside a single cell lies in trapping and integrating a single nanodiamond onto a glass nanopipette filled with carbon electrodes, thereby facilitating its arbitrary 3D positioning within a live cell. This approach enables the first measurement of multiple physical quantities within a live cell under natural conditions, allowing us to observe exactly and locally the direct multiphysiological signals induced by the physiochemical events at the intracellular level.

## RESULTS AND DISCUSSION

**Figure 1.**
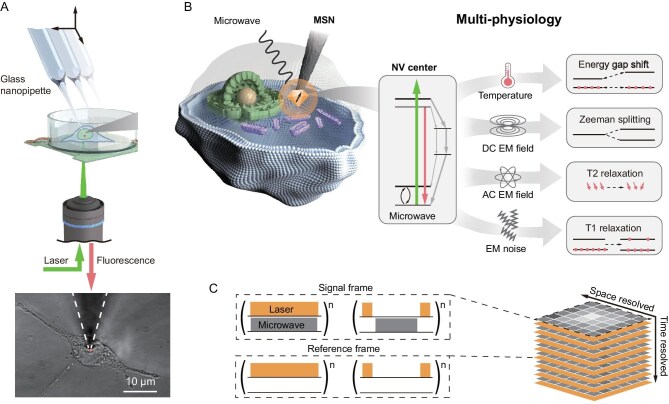
‘Quantum patch clamp’ for live cell quantum multiphysiology. (A) Top: an illustration of the integrative set-up for the MSN-based position manipulation and quantum measurements. Bottom: a fluorescence image of the MSN probe in a live cell. (B) Conceptual description of live cell quantum multiphysiology using an NV center in the MSN probe. EM: electromagnetic. (C) Sensing sequence of spatiotemporal resolved quantum measurements for live cell quantum multiphysiology.

We started by building a scanning-probe-integrated widefield-imaging set-up and live cell quantum measurements as shown in Fig. 1A. The core components are discussed in Fig. S1 (Supplementary  Data), where an inverted microscope was adopted to collaborate with a 3D manipulable NV center spin sensor in a single nanodiamond based on a glass nanopipette, a spin manipulation system through a planar microwave waveguide, and an environment-controlled biological sample chamber, mounted on a piezo stage for nanoscale position control. With a tip diameter of ∼100 nanometers, the MSN probe (Figs S2 and S3) could readily penetrate through the cell membrane and approach the target site in a live cell (Fig. 1B) while maintaining the cell viability, as in live cell patch-clamp recording. This enables positioning the NV center probe in a live cell and performing *in situ* multiphysical quantum sensing. The quantum multiphysiology scheme is outlined in Fig. 1B. The NV center, usually negatively charged (NV^−^), is a solid-state electron spin defect comprising a substitutional nitrogen adjacent to a vacancy in diamond, which constitutes a spin-1 system [11]. Its spin state can be coherently manipulated by microwave pulses and detected via fluorescence under a laser initialization (532 nm), empowering optical detected magnetic resonance (ODMR) for precision measurements [11,24].

When the NV center in MSN is manipulated in a live cell (Fig. 1B), it interacts locally with the multiphysiological environment, such as temperature [12,25], electromagnetic field [19,26], strain [27] and free radical species [21,28]. As a result, its electron spin properties can be modified, leading to a shift of the zero-field splitting, the field-modulated Zeeman splitting or the acceleration of the longitudinal spin relaxation, which can be captured in NV-based spin spectroscopy [29] and T1 relaxometry. To simultaneously track the position of the NV center and perform intracellular quantum sensing, we designed an imaging sequence to provide spatiotemporal information and signal integration to improve the signal-to-noise ratio (SNR, Fig. 1C and Fig. S4). During each frame, a measurement sequence or reference sequence was used for controlling coherent spin manipulation, while laser initialization and camera readout were run repeatedly and photons carrying information of the measured parameter were accumulated until the exposure ended. This acquisition method eliminates the inevitable background and improves the SNR of ODMR and T1 to 20 and 102, respectively, enabling high-spatiotemporal-resolution quantum sensing.

**Figure 2.**
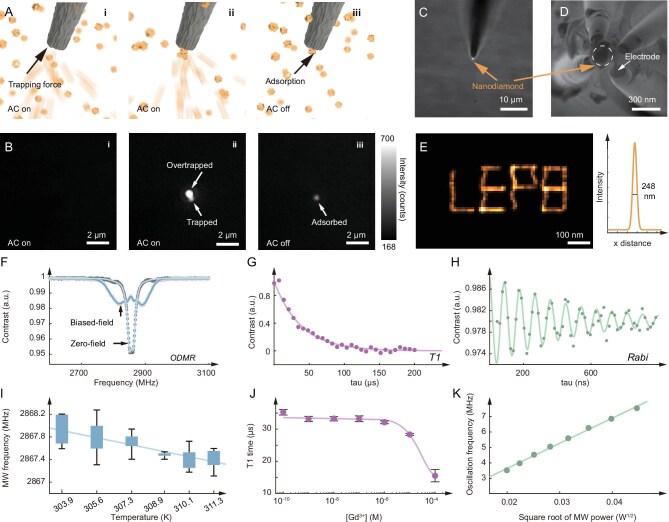
MSN-based NV-center electron spin probe. (A) Single nanodiamond trapping by applying a high-frequency AC electric field to the glass quadrupole nanoelectrode. (B) Fluorescence imaging of the single nanodiamond trapping process. (C) Merged fluorescence and bright field image of the MSN probe. (D) SEM image of a single nanodiamond at the tip of the glass nanoelectrode. (E) Demonstration of the sub-diffraction manipulation of a single nanodiamond (left) and the Gaussian profile of the fluorescence signal of a single nanodiamond (right). The resulting trajectory from the single-molecule tracking algorithm shows the designed pattern ‘LEPB’. (F) ODMR experiment with and without a biased magnetic field by a permanent magnet. (G) T1 relaxometry of the MSN probe in Dulbecco's modifiled eagle medium (DMEM) medium. (H) Rabi oscillation of the MSN probe in air. (I) Temperature dependence of the resonance peak (MW frequency in ODMR) of the MSN probe. MW: microwave. (J) T1 relaxation time as a function of the external electromagnetic noise controlled by Gd^3+^ concentration. (K) Rabi oscillation frequency versus microwave power.

Implementing the concept of MSN requires fabricating a glass nanopipette with only a single nanodiamond at the tip, presenting a significant engineering challenge. Rather than using conventional micro-electro-mechanical-system (MEMS) fabrication, here we utilized a custom-developed single-particle trapping technique [30,31] for controllable electrical trapping of single nanodiamonds directly onto the carbon-electrode-filled glass nanopipette. A four-barrel glass pipette was laser pulled to form nanoscale channels which were then filled by carbon deposition (Fig. S2). We obtained a quadrupole nanoelectrode with a diameter of ∼300 nm. The trapping of a single nanodiamond is achieved using a gradient dielectrophoretic force (Fig. 2A and Fig. S3). To select and trap a nanodiamond, we moved the tip to a target nanoparticle and applied an alternating current (AC) voltage. Nanodiamonds close to the tip are driven to the tip and one target nanodiamond is first trapped electrically (‘trapping mechanism’ in Supplementary Discussion), as shown in Fig. 2A and B. After switching off the trapping voltage, all the nanodiamonds but the targeted one in touch with the nanoelectrode diffused away from the tip. In our experiments, single nanodiamonds can be trapped within a trapping frequency range of 0.8–1.0 MHz with a > 90% success rate, when preparing such probes (‘single nanodiamond trapping’ video in Supplementary Video 1). After trapping, the targeted nanodiamond was adsorbed at the tip instead of freely rotating, which we attributed to the interaction between the nanodiamond and the carbon nanoelectrodes. Electron microscopy analysis (Fig. 2C and D, and Fig. S2) reveals a single nanodiamond with a diameter of ∼100 nm trapped at the center of the four nanoelectrodes, indicating the successful integration of the NV probe to the glass nanopipette.

A notable advantage of the present approach is the active manipulation enabled by the glass nanopipette, whose position relative to the specimen of interest can be controlled using a precision stage. The manipulation precision is demonstrated by moving the probe to navigate a pattern with a step size of 0.4 nm. Employing single-particle localization and tracking [32], we obtained a super-localized image pattern (‘LEPB’, the abbreviation of our lab name), with a localization precision of 4.5 nm (Fig. 2E), indicating the ultrahigh accuracy of our manipulation. This has not been possible with NV-based nanothermometry without position control [12].

To benchmark the quantum sensing performance of the MSN probe, we performed multimodal NV sensing experiments under environment-controlled *in vitro* conditions. The ODMR results in Fig. 2F clearly demonstrate the magnetic sensing capability of the single nanodiamond (Figs S4 and S5). The temperature dependence of the zero-field splitting was obtained by recording ODMR spectra under a set of controlled temperatures (Fig. 2I and Fig. S6), resulting in a temperature dependence of −0.074 kHz/K that aligns with the reported value [25]. Before each experiment, a calibration measurement is first conducted to eliminate the performance heterogeneity of single nanodiamonds [33] (Fig. S6). In addition, NV-based T1 relaxometry can provide information regarding local electromagnetic noise which can be caused by free radicals [21] or paramagnetic ions [34]. We evaluated the T1 response of our MSN probe in a series of Gd^3+^ concentrations (Fig. 2G and J). The result shows that the T1 value decreases with the increase of Gd^3+^ concentration due to the acceleration of the longitudinal spin relaxation. These *in vitro* measurements not only confirm the multimodal quantum sensing capability of this probe in aqueous solution but also offer sensitivity calibrations for the following intracellular measurements. Moreover, we demonstrated coherent quantum control and spin state measurement with the MSN probe. Coherent Rabi oscillation [35] was induced and observed by tuning the microwave frequency, allowing the measurement of coherence time (T2) and its dependence on the AC electromagnetic field (microwave power, Fig. 2H and K and Fig. S7).

As we could now manipulate the NV spin sensor in a ‘patch-clamp’ manner, we applied the MSN probe for quantum sensing in a live cell (Supplementary Video 2, Supplementary Discussion) to directly record the multiphysiological signals from the physiochemical processes. In multiphysiology, the electric field in cell signaling has been well studied in electrophysiology. Nevertheless, it remains challenging to spatiotemporally capture the local heat-induced temperature change and the intracellular free-radical-induced electromagnetic field noise change, which are indicators of intracellular energy conversion and homeostasis processes [36]. We pursue this task of probing local temperature and electromagnetic field noise using the MSN to demonstrate such multiphysiology measurements.

**Figure 3.**
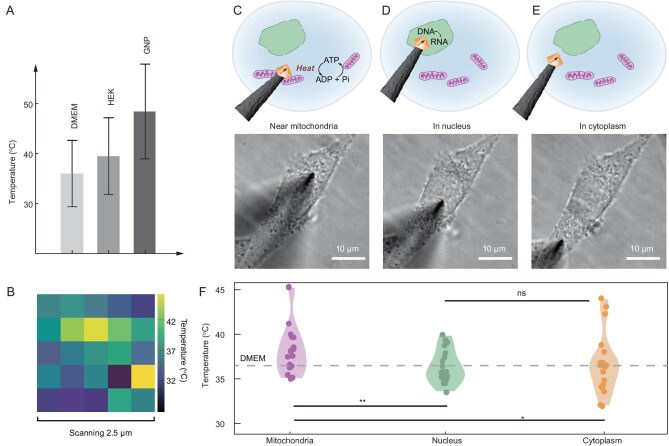
Intracellular temperature measured at different sites. (A) MSN-based nanothermometry in DMEM, single HEK cell, and GNP photoheated single HEK cell. (B) Scanning image of intracellular temperature distribution by the MSN probe in a single HEK cell. (C–E) Illustration of the MSN probe placed at different intracellular sites (top) and corresponding bright field images of the MSN probe placed at different intracellular sites (bottom). (F) Measured intracellular temperature within a single C6 cell by the MSN probe. The significance is calculated from t-test, and the *p*-values are shown as ** for *P* ≤ 0.01, * for *P* ≤ 0.05. ns for no significance.

To modulate the local cellular temperature response, we introduced gold nanoparticles (GNPs) to single human embryonic kidney (HEK) cells, in order to stimulate a photothermal effect [12]. The measurement results show a temperature value of 36.0 ± 6.63°C, 39.5 ± 7.67°C and 48.4 ± 9.54°C, for medium, living HEK cells and GNP photoheated HEK cells, respectively, confirming the intracellular temperature sensing capability (Fig. 3A). By scanning 2.5 μm × 2.5 μm (a small region is selected to diminish cell damage by scanning, Fig. 3B), we demonstrate that the MSN probe can spatially map the local temperature distribution in the cell cytoplasm. We also applied the MSN probe to measure the local temperature at selected sites inside a live cell (Supplementary Video 3). With the same probe, we investigated different target sites including a region with concentrated mitochondria (Fig. 3C and Fig. S8), nucleus (Fig. 3D) and cytoplasm (Fig. 3E). Considering the errors induced by the heterogeneity of live cells and nanodiamonds, and the deteriorated performance of nanodiamond on the tip, we made efforts to improve the accuracy of these measurements, including reducing the noise and filtering probes with bright nanodiamonds (Fig. S4). The results show temperature values of 37.8 ± 2.60°C, 35.8 ± 2.88°C and 36.5 ± 4.90°C as given in Fig. 3F, indicating temperature heterogeneity among intracellular sites. We found that the probed region with concentrated mitochondria displays the highest average temperature. This observation is in accordance with the energy-conversion-powering postulation of mitochondria.

**Figure 4.**
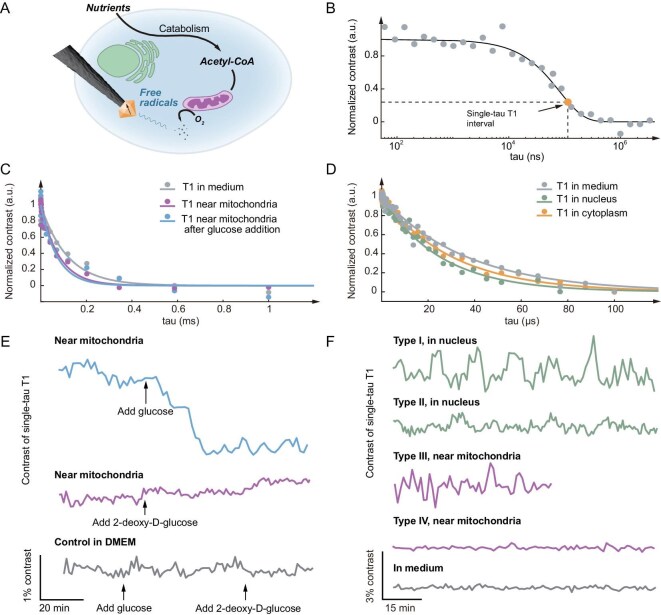
Intracellular free radical dynamics probed by T1 relaxometry. (A) Simplified intracellular pathway of intracellular free radical generation. (B) T1 relaxometry and single-tau T1 relaxometry. (C) T1 relaxometry in a single C6 cell before and after adding glucose in the medium to stimulate cell activities. (D) T1 relaxometry in a C6 cell at different intracellular sites. (E) Real-time T1 traces of the MSN probe in single C6 cells near mitochondria show different tendencies of T1 contrast change when adding glucose and 2-deoxy-D-glucose. (F) Real-time T1 traces of the MSN probe in different sites of a single C6 cell show varied oscillations and fluctuations. The traces in (E) and (F) are moving-averaged and down-sampled from the raw data for improved SNR. The actual sampling interval of the traces is ∼36 s.

To probe intracellular free radical generation, microwave-free T1 measurements (Fig. S9), which maintain cell viability (Supplementary Discussion, Fig. S10) and directly record local electromagnetic field noise dynamics, were also conducted (Fig. 4A). In addition to the spatial resolution for targeting different sites in a single cell, our approach also offers temporal resolution, crucial to unveiling the dynamics of cellular activities. We continuously ran the T1 sequence at a fixed interval instead of sweeping over a set of tau values, known as single-tau T1 relaxometry [37] (Fig. 4B and Fig. S9). This improved the temporal resolution of the T1-based electromagnetic noise measurement to the exposure time used in our set-up, ∼180 ms, sufficient to capture live cell activities. Figure 4C shows relaxation signals obtained in DMEM medium, and cell cytoplasm near mitochondria before and after glucose stimulation, all featuring a single exponential decay. This relaxation process is related to the interaction between the NV center and the external electromagnetic noise that accelerates the spin relaxation process. Faster decay can be seen for intracellular sites than for medium. As in the current case, no paramagnetic ions are involved; T1 change mainly comes from the free radical production in cellular activities. This is confirmed by modulating the intracellular free radical level. We introduced glucose as a nutrient to stimulate free radical generation [38], which led to a further decrease in the T1 value (from 80.0 to 70.7 μs). In turn, 2-deoxy-D-glucose can inhibit intracellular glycolysis [39], resulting in an increase of the T1 value. These stimulation measurements were also performed in single-tau T1 relaxometry as shown in the real-time monitoring trace in Fig. 4E. The dynamic response of single-tau T1 relaxometry further confirms the effective spatiotemporal capture of intracellular electromagnetic noise.

We finally employed T1 relaxometry for pursuing unknown biological findings of native free radical dynamics (Fig. 4D and F, and Fig. S11A), as the present approach can now provide an unprecedented spatiotemporal resolution. Unexpectedly, we observed quasi-periodic oscillations in intracellular environments in time-resolved T1 contrast measurements. The signal peaks are considerably higher than the noise baseline of the silent state recordings. The oscillations occurred occasionally in the nucleus and in the cytoplasm near mitochondria, while displaying different quasi-periodic features. A variety of oscillation and fluctuation phenomena were found within the, nominally, same type of cells (Fig. S11B). Such direct and dynamic free radical observations at single-cell/single-molecule level was not possible using other techniques. Notably, our observations reveal single-site fluctuating events that largely differ from a continuous free radical production expectation. The oscillation feature observed, an indication of biological oscillation, displays signatures of the glycolysis oscillation process [40,41]. The different types of observed phenomena may reflect the rich physiochemical processes executing live cell activities.

## CONCLUSION

Our approach provides a direct approach for delivering NV-center-based electron spin probes to effectively measure a single live cell. The integration of only a single nanodiamond pronounces the nanoscale quantum sensing capabilities by locally manipulating and confining the probe–target interaction space to the biological sample of interest. This advancement enables access to intracellular dynamics and demonstrates live cell quantum multiphysiology, uncovering spatiotemporal insights into temperature heterogeneity and free radical concentration oscillations, which may contribute to novel biological discoveries.

## Supplementary Material

nwaf130_Supplemental_Files
