# Supplementary material for: Live cell quantum multiphysiology enabled by a manipulable single nanodiamond
Source: Natl Sci Rev. 2025 Apr 1;12(6):nwaf130. doi: 10.1093/nsr/nwaf130 (PMC12125982; doi:10.1093/nsr/nwaf130)
Supplement: nwaf130_Supplemental_Files [file nwaf130_supplemental_files.zip › 1777_Supplementary_Information.pdf]

Supplementary Information for

Live cell quantum multiphysiology enabled by a manipulable single nanodiamond

Yang Xu[1], Yibo Yang[1], Yuang Chen[1], Wenxin Zhu[1], Shiyang Lyu[1], Chen Zhang[1], Xingxu Huang[2] and Jiandong Feng[1,2,3]*

[1]*Laboratory of Experimental Physical Biology, Department of Chemistry, Zhejiang University, 310058 Hangzhou, China*
[2]*The First Affiliated Hospital, School of Medicine, Zhejiang University, 310003 Hangzhou, China*
[3]*Institute of Fundamental and Transdisciplinary Research, Zhejiang University, 310058 Hangzhou, China*

**Correspondence should be addressed to* jiandong.feng@zju.edu.cn

Methods

Setup

All experiments were conducted on a home-built inverted microscope equipped with a custom probe module (**Supplementary Fig. 1**) and the live cell sample holder. The live cell sample holder was accurately controlled in the Z-axis using a piezo stage (PI, P 562.3CD) with a displacement precision of 0.4 nm and in the XY-axis using a step stage (PI, V-738.056111) with a minimum displacement of 0.05 μm. A printed circuit board (PCB) on the live cell sample holder delivered the microwave (MW). An acousto-optic modulator (AOM, G&H, AUT-AOM 3080-125) was used to generate laser pulses. An iris blocked all the laser diffraction spots except the secondary diffraction spots. A 532 nm dichromatic mirror (DM, Semrock Di02-R532-25×36) then reflected the incident laser that initialized NV centers into the objective. Subsequently, the DM and a following 635 nm longpass filter (Semrock BLP01-635R-25) filter off most of the reflected laser signal. Finally, the fluorescence signal was detected by a scientific complementary metal-oxide-semiconductor camera (sCMOS, Photometrics Prime 95B). Microwave (MW) signal generated by a MW synthesizer (National Instruments QuickSyn FSW-0020) was transmitted through high-fidelity cables (ST18-SMSM) and amplified by a power amplifier (Mini-Circuits ZHL-16W-43-S+) to several watts. An MW switch (Mini-Circuits ZYSWA-2-50DR+) was used to generate MW pulses. An isolator (Enicom TTG32A10.5Z) was used to protect the amplifier from damage by the reflected MW. Control signals were sent from a pulse signal generator (PSG, SpinCore PulseBlasterUSB) to the AOM, the MW switch, the MW synthesizer, and the sCMOS

for synchronization and pulse sequence encoding. Additionally, the PSG was triggered by the output signal from the sCMOS to further guarantee the synchronization.

Live cell sample holder for live cell multiphysiology using the MSN probe

The live cell sample holder consists of a solution chamber, a temperature modulation module, a custom PCB board, and a planar waveguide. The planar waveguide was patterned on a glass coverslip using photolithography and magnetron sputtering. The outer surface of the solution chamber was covered with a flexible heater connected to the temperature modulation module for high-precision temperature control and achieved temperature feedback by the thermocouple.

Fabrication of the MSN probe

The glass quadrupole nanopipettes were fabricated using a similar approach as detailed in our previous work for fabricating glass multi-pole nanopipettes[1, 2]. A quartz capillary (Φ1.2 mm×0.2 mm×75 mm, Laiyang Zhong Cheng Quartz Glass Co., Ltd.) was laser pulled (P-2000, Sutter Instrument) and butane-blowtorch-heated to fabricate carbon nanoelectrodes. The outside quartz layer was etched in buffered oxide etchant (BOE, 40% NH_4F aqueous solution: 49% HF aqueous solution = 10:1, volume ratio, purchased from Sigma-Aldrich) for three minutes to expose the carbon electrode, which is finally connected to a signal generator (Tektronix, AFG1062).

A suspension of 100 nm nanodiamond (Adamas Nanotechnologies.) was diluted to 0.002-0.01 mg/mL using DI water and sonicated for 30 minutes before trapping. A

trapping electric field was generated by applying a dual-channel square wave signal to trap a single nanodiamond at the tip. (**Supplementary Video 1**)

Quantum measurement using the MSN probe

To illuminate the nanodiamond, the 532 nm laser (~ 100 μW) was focused at the tip of the MSN probe mounted on the custom probe module (**Supplementary Fig. 1**). Meanwhile, the planar waveguide delivered the MW to the nanodiamond. For synchronization among the AOM, the sCMOS, and the MW switch, the PSG provided transistor-transistor logic (TTL) signals.

For the continuous-wave ODMR (cwOMDR) experiment, the laser and the MW continuously initialize and excite NV spin in the signal frame while only the laser initializes NV spin in the reference frame. The signal frame-reference frame pair were repeated 50 times under each MW frequency before switching to the next frequency. The exposure time is set to 3-5 ms according to the fluorescence intensity of the nanodiamond on the MSN probe. The MW frequency was swept from 2835 MHz to 2895 MHz with a 1 MHz step size.

For T1 relaxometry, one T1 measurement consisted of an initializing laser pulse, a readout pulse, and the interval between the two pulses. In the signal frame, the T1 measurement was repeated thousands of times. In the reference frame, the numbers of laser pulses and intervals are equal to those in the signal frame, only all the laser pulses and intervals in the signal frame were combined correspondingly to form a prolonged laser pulse and a prolonged interval, respectively. To record the complete exponential

curve, the interval time between the initializing laser pulse and the readout laser pulse was swept from 40 ns to 1 ms. At each interval time, 50 signal frame-reference frame pairs were recorded. To record a real-time T1 trace, the interval time was fixed to a selected value.

In Rabi oscillation, the reference frame is the same as the signal frame in T1 relaxometry, while the signal frame applies microwave pulses for spin manipulation in the interval between the initializing and readout laser pulses compared with the reference frame.

Intracellular experiment using the MSN probe

Cells were cultured in cell culture medium (DMEM: FBS: PS = 45:5.5:0.5, Shanghai Sangon Biotech. Co.) and incubated on a planar waveguide overnight before the experiment. The multi-physical parameters dependence of NV center spin properties was calibrated in the cell culture medium before measuring intracellular signals for each MSN probe. The MSN probe was lifted when searching for region of interest (ROI) and lowered to insert into the cell for intracellular measurements. The MSN probe was lifted from the cell during switching of sites, to avoid possible mechanical damage to the cell. In stimulation experiments, glucose solution and 2-deoxy-D-glucose were added to the live cell sample holder to reach a final concentration of 13.5 g/L and 10 mM, respectively.

Supplementary Figures

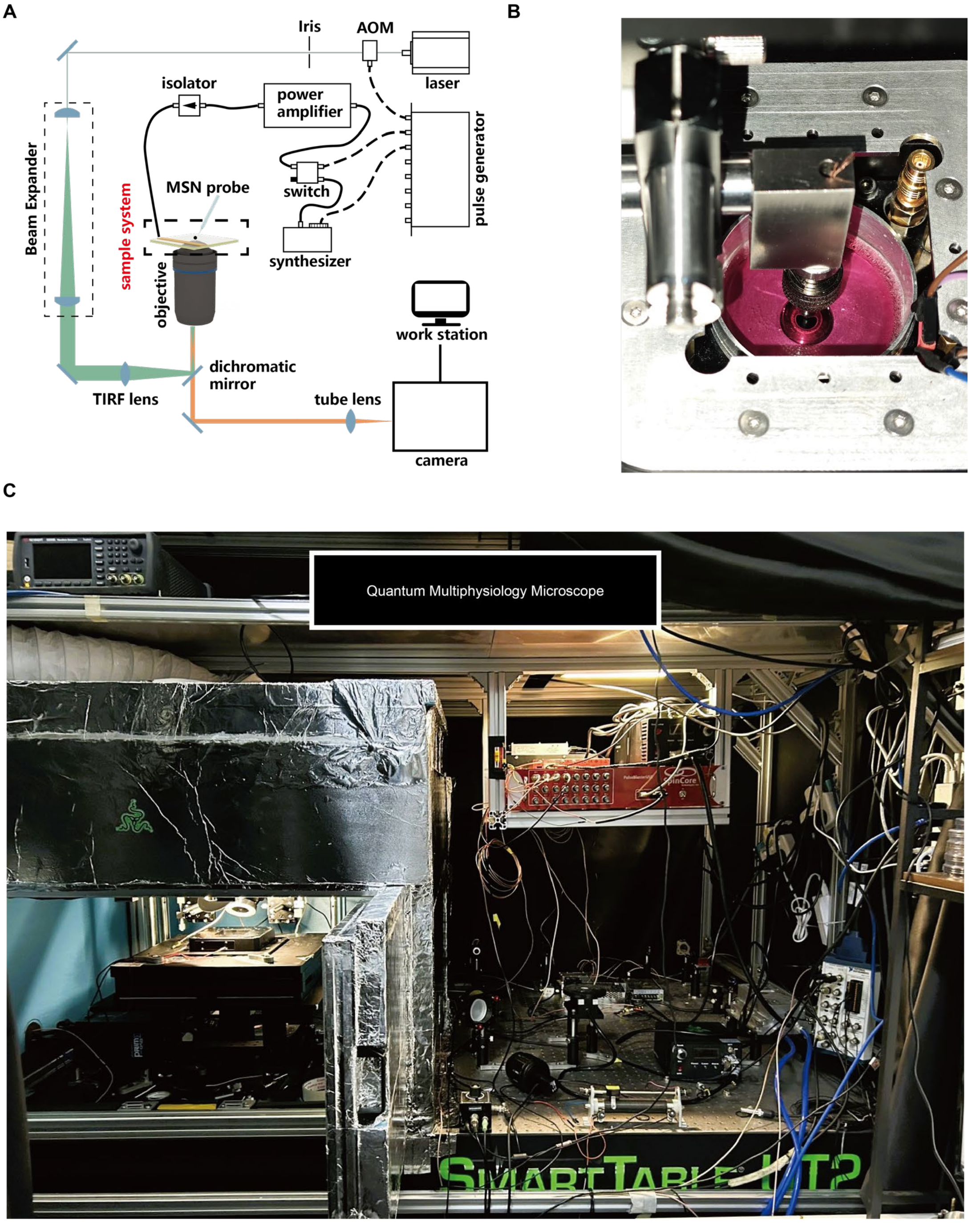

Supplementary Figure 1. Setup of the MSN probe enabled quantum multiphysiology microscope.

A. Schematic view of the setup. Orange beams and green beams stand for fluorescence and laser, respectively. Black curves and dashed curves mark MW signal pathways and control signals, respectively. The live cell sample holder was omitted. **B.** Custom probe

module for manipulating the MSN probe and live cell sample holder for measuring live cell multiphysiology. **C.** The quantum multiphysiology microscope.

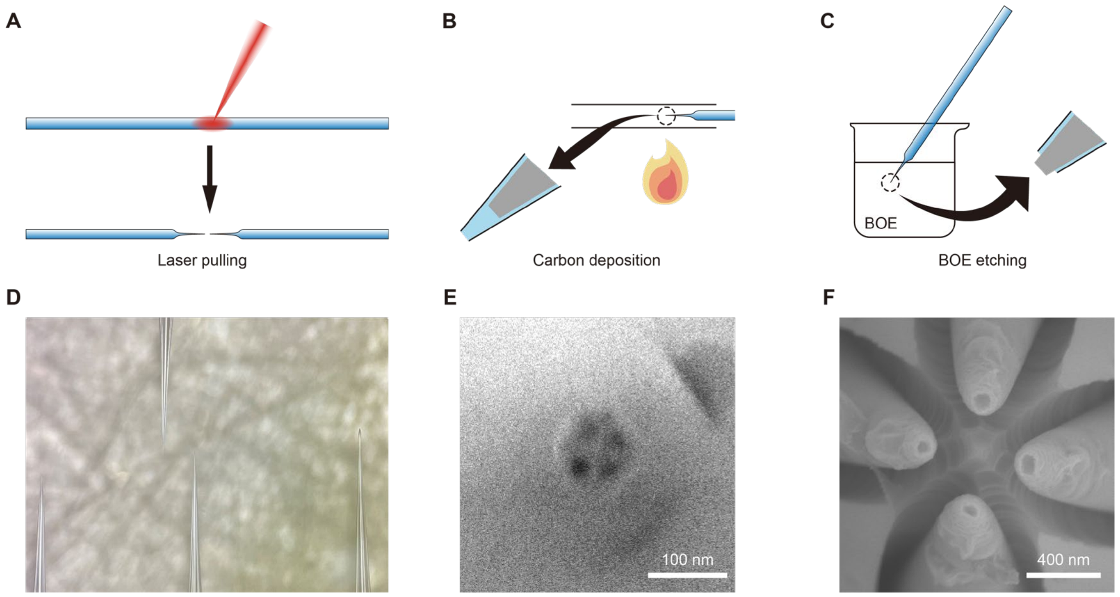

Supplementary Figure 2. Workflow of the MSN probe fabrication and characterization.

The fabrication of the MSN probe consists of three main steps: **A.** Laser pulling of quartz capillary to form nanopipettes. **B.** Carbon deposition to form nanoelectrodes. **C.** BOE etching to remove quartz layer covering carbon electrodes. **D.** Optical image of the glass nanopipettes shows four identical glass nanopipettes pulled. **E.** SEM of plain glass nanopipettes. The tip diameter is 108 nm. **F.** SEM of etched carbon nanoelectrodes where the interval between electrodes is ~400 nm at maximum and the diameter of the electrode is less than 100 nm.

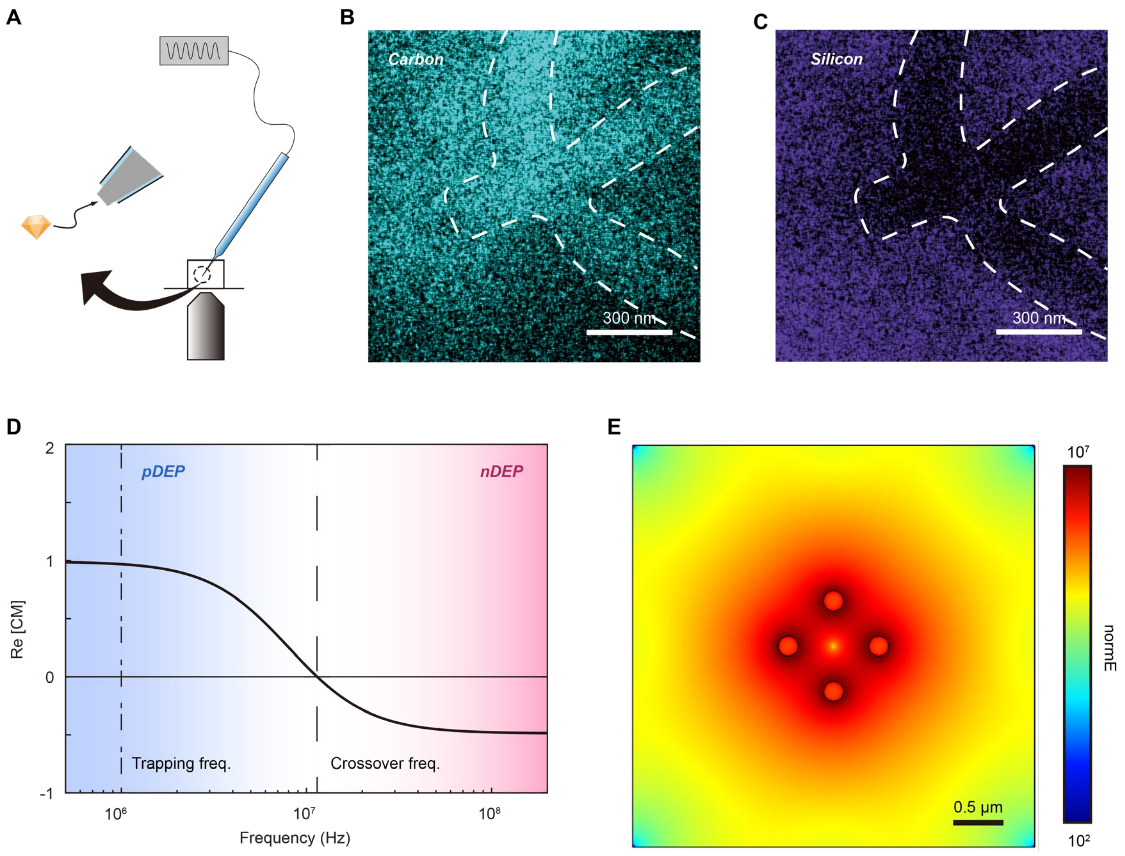

Supplementary Figure 3. Trapping nanodiamonds using a high-frequency electric field.

A. Trapping nanodiamond by applying high-frequency AC signal. **B.** The distribution of carbon in the energy dispersive X-ray spectroscopy (EDS) mapping conforms with the combined shape of the nanodiamond and the four nanoelectrodes. **C.** The distribution of silicon in the EDS mapping is in accordance with the outer quartz layer, proving that the object in the center of the SEM image is nanodiamond. The EDS of the MSN probe in **Fig. 2D** shows a complementary distribution of carbon and silicon. **D.** The trapping frequency (dotted dashed line) is much lower than the crossover frequency (dashed line), indicating nanodiamonds can be trapped in the pDEP region. **E.** The distribution of the electrical field at the tip of the MSN probes indicates the

probability of nanodiamond trapping reaches maxima at intervals between every two adjacent electrodes for the pDEP mechanism where the intensity of the electric field is the highest while at the center of four electrodes for Paul trap mechanism where the intensity of the electric field is the minima.

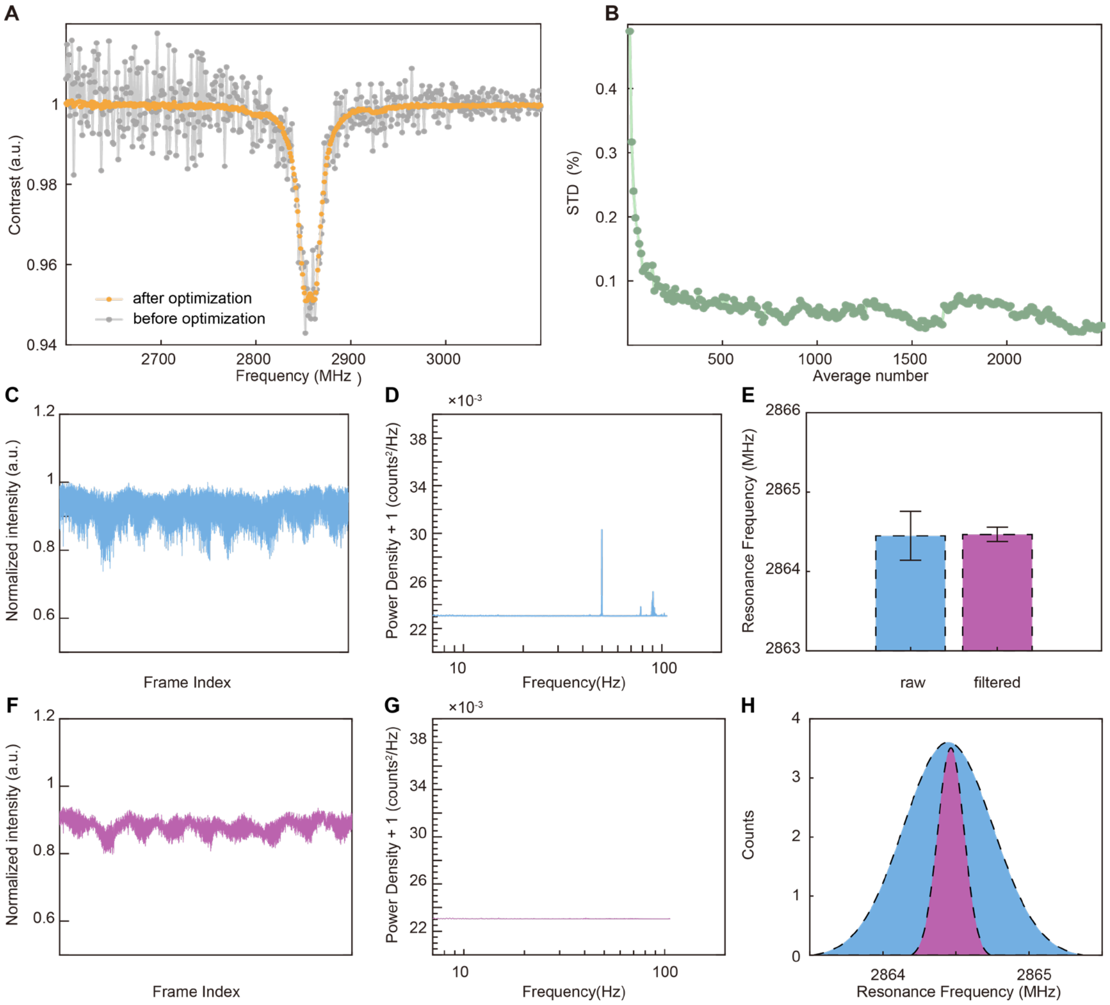

Supplementary Figure 4. Optimization of SNR in ODMR.

A. The original ODMR spectrum of a nanodiamond on an MSN probe before optimization is severely deteriorated compared to the optimized ODMR spectrum. **B.** The standard deviation (STD) of the ODMR spectrum is negatively correlated to the increase of data averaging number. When the averaging number reaches 50, STD is lower than 0.1%, which means an SNR of more than 20 given the ODMR contrast being ~2%. **C.** Time trace of ten consecutive ODMR experiments. **D.** The power spectrum density (PSD) of the time trace shows three peaks at 50 Hz, 80 Hz, and 90 Hz, which we attribute to typical frequencies of noise (electricity, fan-induced fluctuation, random thermal fluctuation). **E.** The resonance frequency extracted before and after applying

notch filters remains unchanged while the confidence interval narrows. By applying notch filters, **F.** The bandwidth of the time trace was narrowed and **G.** The noise peaks were eliminated. **H.** Comparing the distribution of fitted resonance frequency of the ten ODMR experiments before and after signal filtering, we found that the FWHM of resonance frequency distribution decreased from 0.71 MHz to 0.21 MHz, indicating that the performance of the ODMR measurement with the MSN probe can be largely improved.

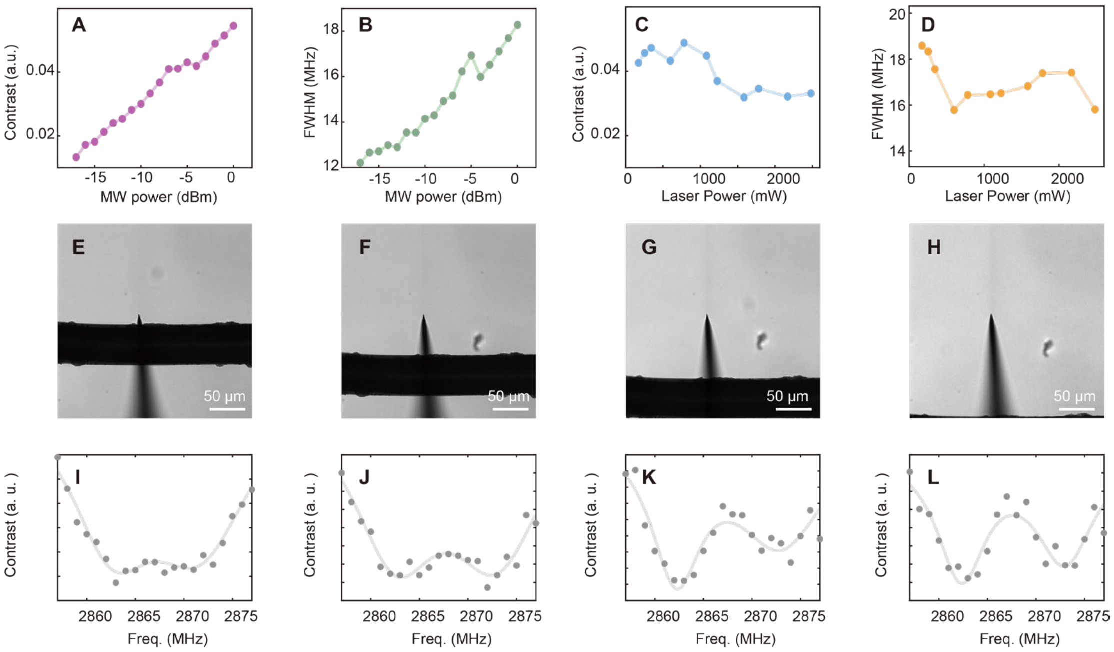

Supplementary Figure 5. Influence of MW power on ODMR.

A. ODMR contrast is positively correlated to the increase of MW power. **B.** The full width at half maxima (FWHM) of ODMR resonance peaks is positively correlated to the increase of MW power. **C.** ODMR contrast decreases slightly with the increase of laser power. **D.** The FWHM of the ODMR resonance peaks remains the same with the increase of laser power. **E-H.** Bright-field images of the MSN probe and the planar waveguide with different tip-waveguide distances. **I-L.** ODMR spectra corresponding to **E-H** show decreasing in FWHM with the increase of tip-waveguide distance, suggesting the decrease of radiated MW power. By balancing the quality ODMR signal and MW radiation power, we can achieve good sensitivity while reducing MW damage to live cells.

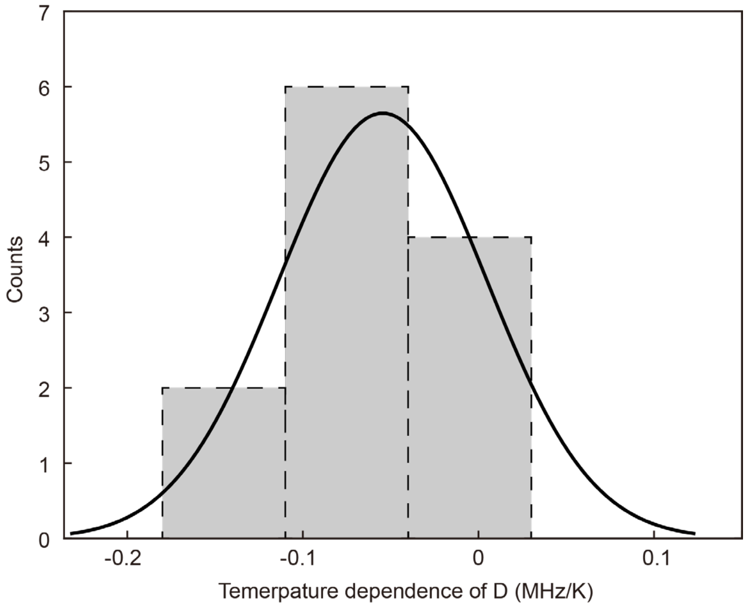

Supplementary Figure 6. Temperature dependence of resonance frequency of the MSN probe.

As shown in **Fig. 2I**, the temperature dependence of resonance frequency of the MSN probe displays a linear relationship. The distribution of 12 different MSN probes shows the expectation of temperature dependence of resonance frequency is 0.056 K/MHz.

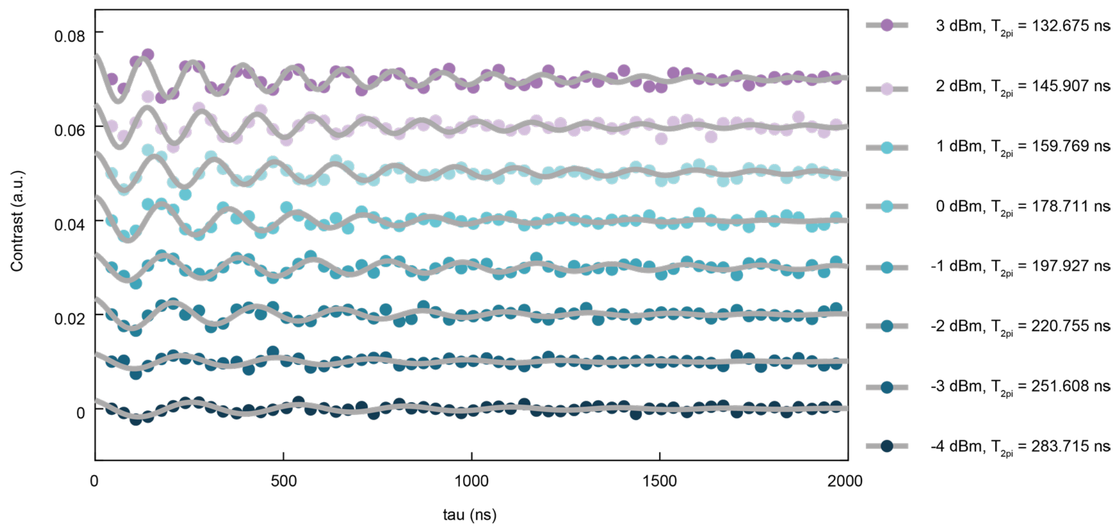

Supplementary Figure 7. Rabi oscillations of the MSN probe under different MW power.

As shown in **Fig. 2K**, the Rabi oscillation frequency is proportional to the square root of microwave power. Here are the oscillation curves corresponding to the data points in **Fig. 2K**. In addition, the Rabi contrast increases with the increase of MW power.

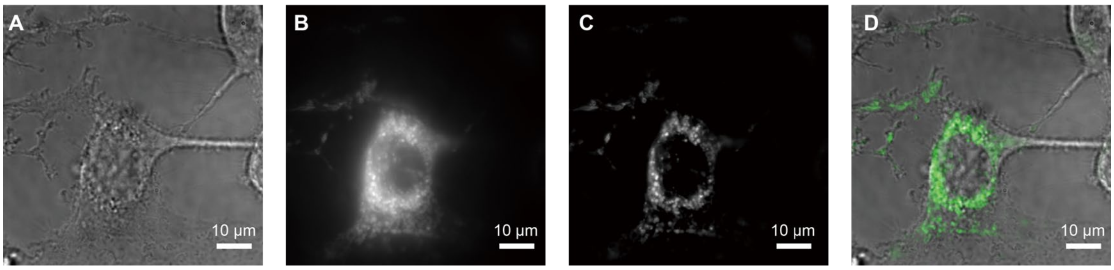

Supplementary Figure 8. Localization of mitochondria in C6 cells.

Correlating the bright field image of C6 cells (**A**) and the fluorescence image of labeled mitochondria in C6 cells (**B**) indicates the observed concentrated mitochondria region under the bright field is in good accordance with the fluorescence image. By applying a Gaussian filter (**C**) of the original fluorescence image (**D**), unwanted autofluorescence and off-focus fluorescence are eliminated, resulting in a fluorescence image with high SNR.

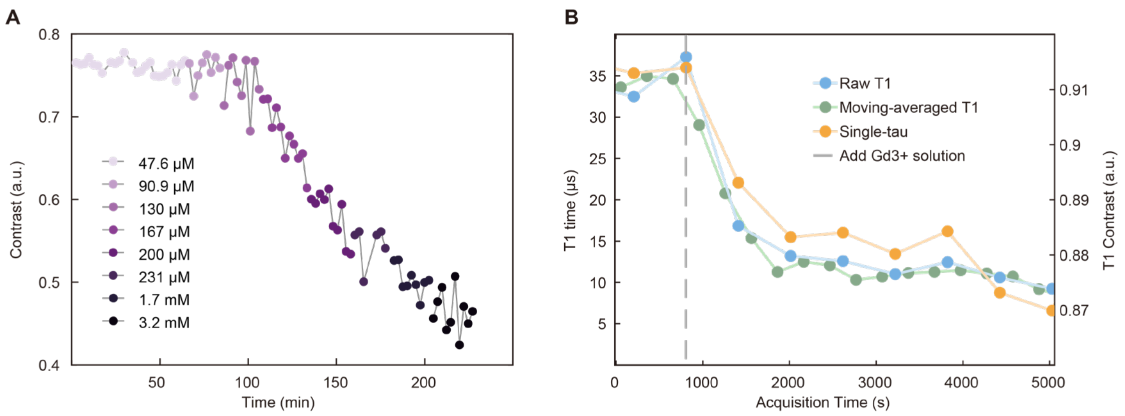

Supplementary Figure 9. Verifying single-tau T1 relaxometry and single-tau T1 relaxometry.

Real-time T1 trace is obtained by single-tau T1 relaxometry. Instead of sweeping the full T1 spectrum, single-tau T1 relaxometry monitors the T1 contrast at a fixed interval tau. **A.** Real-time T1 traces of the MSN probe in Gd^{3+} solution (from light purple dots to dark purple dots, the concentration of Gd^{3+} increases). **B.** Comparison among results of T1 relaxometry, moving-averaged T1 relaxometry, and single-tau T1 relaxometry indicates that single-tau T1 relaxometry can also reflect the change of external EM noise through T1 contrast.

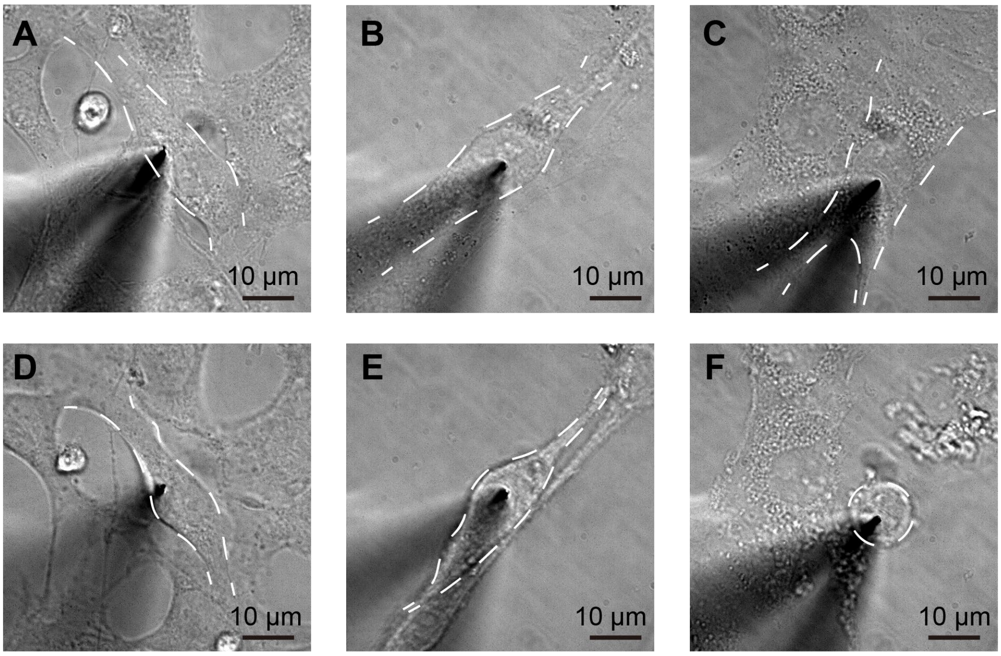

Supplementary Figure 10. Cell structures before and after measurement.

Bright-field images of C6 cells maintained shape (**A, D**), slightly changed shape (**B, E**), and significantly changed or lost shape (**C, F**) after performing single-tau T1 relaxometry for more than 2 hours. Most of the cells can maintain their shapes as in (**A, D**).

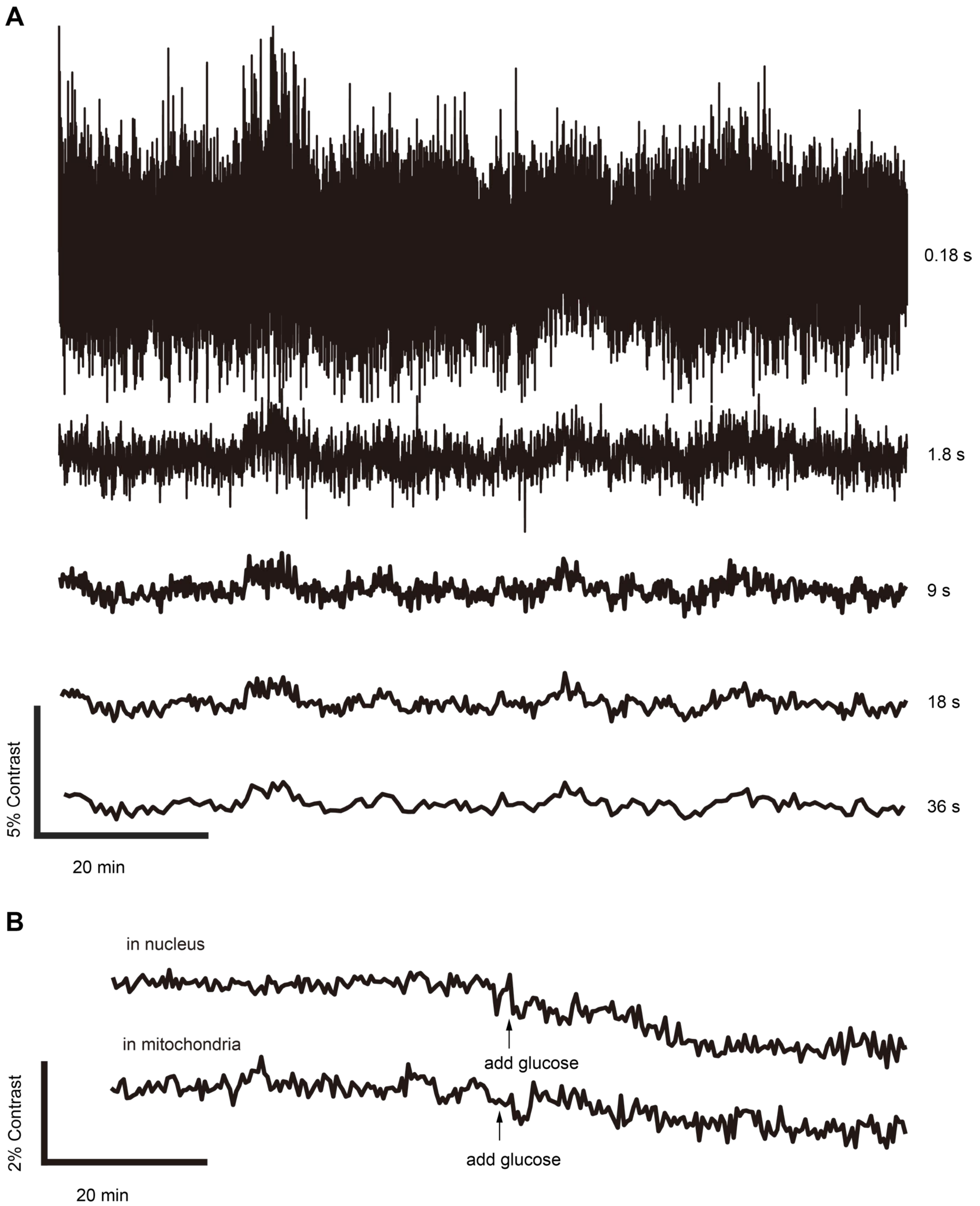

Supplementary Figure 11. Data moving-averaged and down-sampled and electromagnetic noise dynamics in C6 cells.

(**A**) Raw trace in Fig. 4F down-sampled for improved SNR. (**B**) Real-time T1 traces measured after adding glucose.

Supplementary Discussion

Single nanodiamond trapping mechanism

For the single nanodiamond trapping mechanism by AC electrical field, both dielectrophoresis (DEP)[1] and Paul trap[3] contribute to the trapping process.

We calculated the crossover frequency of negative DEP (nDEP) and positive DEP (pDEP) according to the Clausius-Mossoti factor[4] by

$$\left(\frac{1}{2\pi}\right)\left[\frac{(\sigma_p-\sigma_m)(\sigma_p+2\sigma_m)}{(\epsilon_m-\epsilon_p)(\epsilon_p+2\epsilon_m)}\right]^{\frac{1}{2}}, \quad (1)$$

where σ_p and σ_m are the conductivity of particle and medium, respectively, and ϵ_p and ϵ_m are the permittivity of particle and medium, respectively. Using the values in **Supplementary Table 1**, the crossover frequency is calculated to be 11.47 MHz, indicating a contribution of the pDEP mechanism[3] compared with the trapping frequency of 0.8~1 MHz (**Supplementary Fig. 3**). In the pDEP mechanism, particles are trapped at the maxima of the local electric field. Considering that the particle size of nanodiamonds is close to the tip diameter of the MSN probe, the four electric field maxima at intervals between adjacent electrodes would produce a resultant force that attracts nanodiamonds to the center of the four electrodes.

In addition, we simulated the distribution of the local electric field of the square wave (1 MHz, 5Vpp) at the tip of the MSN probe (**Supplementary Fig. 3**). In the Paul trap mechanism, the trapping site is at the minimum of the local electric field. The electric field minima at the center of the four electrodes will produce a force that also

attracts nanodiamonds to the center of the four electrodes, indicating a contribution of the Paul trap effect.

Performance of the MSN probe

The localization resolution

The localization resolution of the MSN probe refers to the localization precision of the MSN probe in its fluorescence readout. The ~4.5 nm localization precision of the MSN probe we used is credited to the brightness of the nanodiamond, the stiffness of the MSN probe, and the stability of the setup. Given that the setup performance is fixed, further improvement in the localization resolution of the MSN probe can be made by employing nanodiamonds with smaller particle sizes and higher NV density and fabricating MSN probes with higher stiffness.

The temporal resolution

To improve the temporal resolution of T1 relaxometry, we adopted the single-tau sensing protocol to observe the real-time intracellular dynamics by taking advantage of the monotonicity of single exponential decay. As shown in **Supplementary Fig. 8**, the single-tau T1 curve aligns with the full T1 spectrum, proving the feasibility of single-tau relaxometry. Given that a full T1 spectrum consists of 30-50 data points, the temporal resolution of single-tau relaxometry was improved 30-50 times compared to T1 relaxometry.

The long-term intracellular observation of the living cell

As shown in **Supplementary Video 2**, cells in the environmental-controlled live cell sample holder maintained a normal physiological state for several hours, and cell division was also observed, indicating maintaining normal cell activities.

Due to the microwave effect in continuous observation (≥5 minutes) using ODMR, ~33.3% of the cells maintained their original shapes after ODMR experiments. In contrast, 88.2% of the cells maintained their original shape after T1 relaxometry, indicating reduced cellular damage. Thus, we chose T1 relaxometry to observe real-time intracellular multiphysiology. In single-tau T1 relaxometry, there are over half of the cells maintained their shapes even after the observation of more than 2 hours. From the result, single-tau T1 relaxometry not only provides high temporal resolution but also causes low cell damage that benefits the observation of the dynamics of intracellular multiphysiology.

Multi-physical sensing

NV center is sensitive to multiple physical parameters such as magnetic field, temperature, electric field, stress, etc. As electrophysiological signal has already been well studied by the patch-clamp technique. We have demonstrated the measurement of the two most important parameters in cell multiphysiology apart from electrophysiological signals: temperature and electromagnetic noise.

Despite the extraordinary magnetic sensing capability of NV centers, there are no considerable changes in DC magnetic fields in the typical cells. In some particular

situations, DC magnetic field in magnetic-related biosamples like magnetotactic bacteria[5] becomes relevant which can also be captured using the MSN probe due to its DC magnetic field sensing capability as shown in **Fig. 2F**.

In addition, the measurement of intracellular stress and strain suffers from the problem of current insufficient sensitivity[6]. A potential solution is to further improve the sensitivity of the NV center by designing new sensing sequences and optimizing the performance of the fabricated NV centers.

Supplementary Table

Supplementary Table 1. Values of parameters for calculating crossover frequency.

Parameters	Values
$\boldsymbol{\sigma_m}$	$200\ \mu S/m$
$\boldsymbol{\epsilon_m}$	$78.5\epsilon_0$
$\boldsymbol{\sigma_p}$	$0.07\ S/m$ [7, 8]
$\boldsymbol{\epsilon_p}$	$1.31\epsilon_0$ [9]

Supplementary Video Captions

Supplementary Video 1.

MSN probe fabrication by single nanodiamond trapping. The nanodiamond was trapped by applying AC signals (square wave, 1 MHz, 5 Vpp) to the carbon electrodes.

Supplementary Video 2.

Long-term cell culture in the live cell sample holder. HEK cells were cultured on the planar waveguide mounted on the live cell sample holder in a cell culture medium at 37 ^{o}C. The movement and division of cells indicate good cell viability using the live cell sample holder.

Supplementary Video 3.

Targeting different sites for intracellular quantum measurements. The MSN probe was moved to the intracellular sites of the C6 cell and then the live cell sample holder was lifted to penetrate the cell for live cell quantum multiphysiology.

Supplementary References

1. Jiang, X.W., Zhou, Y., Chen, Y., Shao, Y.H. & Feng, J.D. Etching-engineered low-voltage dielectrophoretic nanotweezers for trapping of single molecules. *Anal. Chem.* **93**, 12549-12555 (2021).
2. Yuang Chen et al., Programmable Electric Tweezers. arXiv preprint, arXiv:2503.01554 (2025).
3. Guan, W.H., Joseph, S., Park, J.H., Krstic, P.S. & Reed, M.A. Paul trapping of charged particles in aqueous solution. *Proc. Natl. Acad. Sci. U. S. A.* **108**, 9326-9330 (2011).
4. Pohl, H.A. Dielectrophoresis: The Behavior of Neutral Matter in Nonuniform Electric Fields. (1978).
5. Uebe, R. & Schüler, D. Magnetosome biogenesis in magnetotactic bacteria. *Nat. Rev. Microbiol.* **14**, 621-637 (2016).
6. Knauer, S., Hadden, J.P. & Rarity, J.G. In-situ measurements of fabrication induced strain in diamond photonic-structures using intrinsic colour centres. *Npj Quantum Inform.* **6**, 50 (2020).
7. Brown, N. & Hod, O. Controlling the Electronic Properties of Nanodiamonds via Surface Chemical Functionalization: A DFT Study. *J. Phys. Chem. C* **118**, 5530-5537 (2014).
8. Stehlik, S. et al. Electrical and colloidal properties of hydrogenated nanodiamonds: Effects of structure, composition and size. *Carbon Trends* **14**, 100327 (2024).
9. Batsanov, S.S. et al. Giant dielectric permittivity of detonation-produced nanodiamond is caused by water. *J. Mater. Chem.* **22**, 11166-11172 (2012).
